# Comparative Genomic Analysis of Agarolytic *Flavobacterium faecale* WV33^T^

**DOI:** 10.3390/ijms231810884

**Published:** 2022-09-17

**Authors:** Jun Ho Lee, Seong-Rae Lee, Sejong Han, Pyung Cheon Lee

**Affiliations:** 1 Department of Molecular Science and Technology, Ajou University, Suwon 16499, Korea; 2 Department of Polar Sciences, University of Science and Technology, Incheon 21990, Korea

**Keywords:** agarase, CAZymes, *Flavobacterium faecale*, comparative genomics

## Abstract

*Flavobacteria* are widely dispersed in a variety of environments and produce various polysaccharide-degrading enzymes. Here, we report the complete genome of *Flavobacterium faecale* WV33^T^, an agar-degrading bacterium isolated from the stools of Antarctic penguins. The sequenced genome of *F. faecale* WV33^T^ represents a single circular chromosome (4,621,116 bp, 35.2% G + C content), containing 3984 coding DNA sequences and 85 RNA-coding genes. The genome of *F. faecale* WV33^T^ contains 154 genes that encode carbohydrate-active enzymes (CAZymes). Among the CAZymes, seven putative genes encoding agarases have been identified in the genome. Transcriptional analysis revealed that the expression of these putative agarases was significantly enhanced by the presence of agar in the culture medium, suggesting that these proteins are involved in agar hydrolysis. Pangenome analysis revealed that the genomes of the 27 *Flavobacterium* type strains, including *F. faecale* WV33^T^, tend to be very plastic, and *Flavobacterium* strains are unique species with a tiny core genome and a large non-core region. The average nucleotide identity and phylogenomic analysis of the 27 *Flavobacterium*-type strains showed that *F. faecale* WV33^T^ was positioned in a unique clade in the evolutionary tree.

## 1. Introduction

The genus *Flavobacterium* consists of approximately 100 species isolated from diverse environmental sources, such as fresh water, sea ice, soil, and sediments [1]. Some species in the genus *Flavobacterium* have attracted interest for their ability to produce valuable enzymes that can be utilized as biocatalysts in bioremediation or wastewater treatment [2,3]. In addition, some species synthesize carbohydrate-active enzymes (CAZymes), including agarases [4], cellulases [5], and xylanases [6]. These enzymes are widely used as biocatalysts for the bioproduction of biofuels and biochemicals from renewable sources. In particular, the utilization of agar, one of the most abundant polysaccharides in nature and a major component in the cell walls of red algae, has attracted interest in the cosmetic, pharmaceutical, and food industries [7,8]. Agar comprises two main polysaccharides: agarose and agaropectin [9]. Agarose is formed by the repetition of β-d-galactose and 3,6-anhydro-α-L-galactose. Agaropectin has the same basic building blocks as agarose, but the hydroxyl groups of the 3,6-anhydro-α-L-galactose units are partially substituted by sulfoxy, methoxy, or pyruvate residues [10]. Agarases are a group of glycoside hydrolases that digest agar into diverse oligosaccharides, and are classified into α- and β-agarases according to their cleavage patterns [11]. The α-agarases cleave the α-1,3 linkages of agarose to form agaro-oligosaccharides, whereas β-agarases cleave the β-1,4 linkages of agarose to produce neoagaro-oligosaccharides [12]. Phylogenetically diverse marine bacteria, isolated mostly from seawater and marine sediments, including *Alteromonas* [13], *Pseudoalteromonas* [14], *Pseudomonas* [15], and *Vibrio* [16], have been reported to produce agarases with diverse catalytic activities and biotechnological application potentials [17].

In the previous study, we isolated and characterized a novel species, *Flavobacterium faecale* WV33^T^, from the stools of Antarctic penguins [18]. Notably, *F. faecale* WV33^T^ showed agarolytic activity on agar plates, indicating the presence of agarase-encoding genes. As there is no available genome information for the *F. faecale* WV33^T^-type strain, sequencing its genome is essential to clone and characterize a variety of CAZymes, including agarases, at the molecular level, thus enriching our understanding of the abundance and distribution of CAZymes in metabolically diverse *Flavobacterium* strains. In addition, phylogenomics and pan-genomic analyses [19,20] will uncover the evolutionary information and biotechnological potential of *F. faecale* WV33^T^. Here, we describe the complete genome sequence of the agarolytic bacterium, *F. faecale* WV33^T^. Based on genome annotation analysis, seven putative genes encoding agarases were identified and their transcriptional expression was analyzed using quantitative RT-PCR. Phylogenomics, average nucleotide identity (ANI), and pan-genomic analyses of the 27 representative genomes of *Flavobacterium* type strains, including *F. faecale* WV33^T^, were also performed.

## 2. Results

### 2.1. Genome Assembly of Flavobacterium Faecale WV33^T^

**Figure 1:**
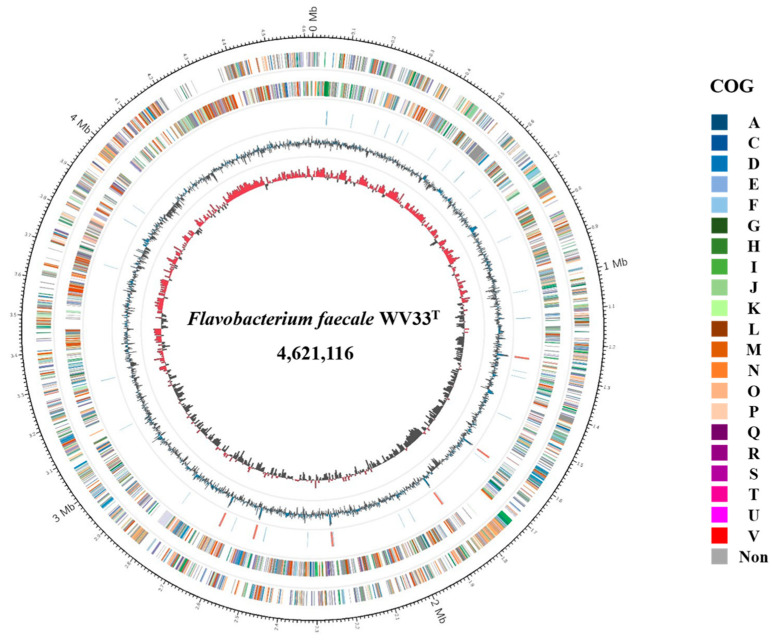
Circular representation of the genome of *F. faecale* WV33^T^. From the outer to inner circle: predicted protein-coding sequences (colored by COG categories) on the plus strand, predicted protein-coding sequences (colored by COG categories) on the minus strand, RNA genes (tRNAs, blue; rRNAs, red), GC content (blue/black), and GC skew (red/black). NC in color codes of COG represents no classified category.

We determined that the genome of *F. faecale* WV33^T^ consisted of a 4,621,116 bp (4.6 Mbp) circular chromosome with a G + C content of 35.2% (Table 1). In total, 3984 coding DNA sequences (CDSs) were predicted, along with 18 rRNA and 67 tRNA genes, resulting in a gene density of 885 genes/Mb (Figure 1). The identified 2573 CDSs in the genome were classified into functional categories based on the clusters of orthologous genes (COG) designation [21] (Table 2) and are presented in the circular map with color codes (Figure 1). The most abundant COG category of genome, except for [S] Function unknown (232 CDSs, 9.0%), was [R] General function prediction alone (289 CDSs, 11.2%), followed by [M] Cell wall/membrane/envelope biogenesis (260 CDSs, 10.1%), [P] inorganic ion transport and metabolism (205 CDSs, 8.0%), [L] Replication, recombination, and repair (183 CDSs, 7.1%), [G] Carbohydrate transport and metabolism (183 CDSs, 7.1%), and [E] Amino acid transport and metabolism (170 CDSs, 6.6%). Notably, similar to the other *Flavobacterium* strains [22,23,24], the high abundance of two COG categories ([G] Carbohydrate transport and metabolism and [E] Amino acid transport and metabolism) suggests evolutionary adaptation of *F. faecale* WV33^T^ to the decomposition of the diverse carbohydrates and proteins present in the uniform environmental niches.

### 2.2. Mining for Gene Encoding Agarases

**Figure 2:**
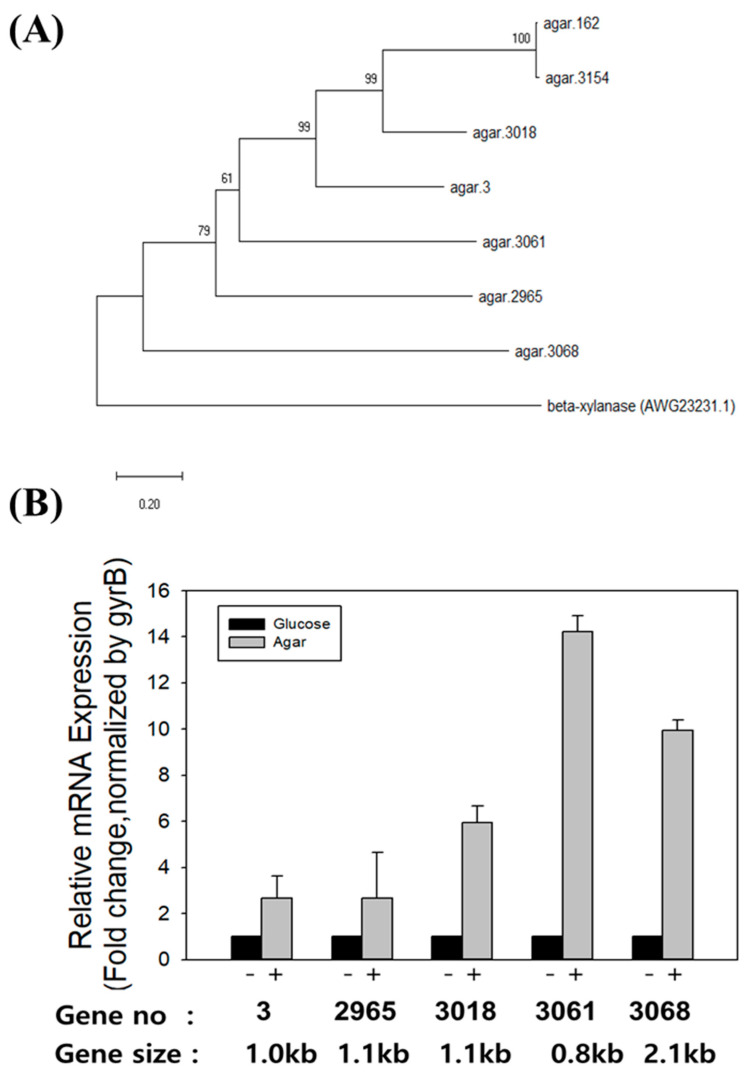
Phylogenetic tree of the seven putative agarases encoded in *F. faecale* WV33^T^ genome and mRNA expression analysis of five of them. (**A**) A multiple alignment performed with ClustalW was used for the construction of a phylogenetic tree through the neighbor-joining method using MEGA-X. Scale bar indicates 0.2 amino acid substitutions per site. Numbers at nodes indicate bootstrap percentages (from 1000 bootstrap replicates). Beta-xylanase of *F. faecale* WV33^T^ (AWG23231.1) was used as an outgroup to root the tree. (**B**) After *F. faecale* WV33^T^ was grown in 100 mL flasks containing 1 g/L sliced solidified agar as carbon source, mRNA expression levels of five putative agarase genes were determined via RT-qPCR. Relative mRNA expression was normalized based on that of the housekeeping gyrB gene (as a control). Bars represent the mean, whereas the error bars represent the standard deviation. Experiments were performed in biological triplicates (*n* = 3).

The presence of genes encoding agarases was first investigated in the complete genome of *F. faecale* WV33^T^ since *F. faecale* WV33^T^ showed agarolytic activity. Seven putative genes encoding agarases (agar.3, agar.162, agar.2965, agar.3018, agar.3061, agar.3068, and agar.3154; see sequences in Figure S1) were identified in the genome of *F. faecale* WV33^T^. The amino acid sequence analysis of these putative agarases revealed that agar.162 and agar.3154 were closely related (forming one cluster), whereas agar.3068 was phylogenetically the farthest from them (Figure 2A). Next, the mRNA expression of the putative agarase genes was investigated under inducible conditions (agar vs. glucose-supplemented media). As the two nucleotide sequences of the agar.162 and agar.3154 genes were highly similar (a local similarity of 99% and a global similarity of 77%) and thus hampered the gene-specific primer design (Table S1) for evaluating individual gene mRNA expression, agar.162 and agar.3154 were excluded from the mRNA expression analysis. Quantitative RT-PCR (RT-qPCR) analysis revealed that the mRNA levels of all five putative agarases were significantly induced, but to different degrees, by the presence of agar in the medium compared to the expression observed when grown in a medium containing glucose (Figure 2B). In particular, the mRNA expression level of agar.3061 was 14-fold higher in the medium containing agar than in the medium containing glucose.

### 2.3. CAZymes of Flavobacterium Faecale WV33^T^

Since *Flavobacterium* strains are known to have diverse carbohydrate-active enzymes (CAZymes), we predicted CAZymes in the genome of *F. faecale* WV33^T^ against the dbCAN database [25], using three CAZyme annotation programs (HMMER, eCAMI, and DIAMOND). The results of the CAZyme analysis revealed that the genome of *F*. *faecale* WV33^T^ contained 154 genes encoding CAZymes (Table 3) predicted by all three annotation programs (high accuracy): the most abundant CAZyme was glycoside hydrolase (GH, 88), followed by glycosyltransferase (GT, 48), polysaccharide lyases (PL, 10), carbohydrate-binding module (CBM, 5), and carbohydrate esterase (CE, 3), accounting for 33 CAZyme genes per Mbp in the genome. Notably, these high numbers of the predicted CAZymes correlated with the number of COG functional categories ([G] Carbohydrate transport and metabolism, 183). When the CAZymes predicted by one or two annotation programs were included (low accuracy), the total number of CAZymes reached 311.

### 2.4. ANI Analysis of the 27 Representative Genomes of Flavobacterium Strains

**Figure 3:**
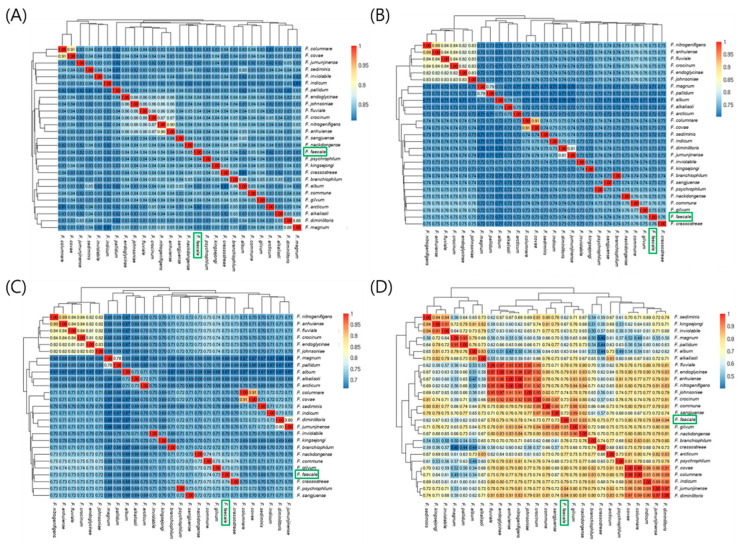
Average nucleotide identity (ANI) analysis of the 27 representative genomes of *Flavobacterium* strains including *F. faecale* WV33^T^. ANI analysis was performed using pyani with ANIm (**A**), ANIb (**B**), ANIblastall (**C**), and TETRA (**D**) algorithms. ANI similarities with 1 being identical are indicated in the heatmaps. Scale of similarity values are represented by a continuous color gradient. A similarity value is presented in each cell. Green box represents *F. faecale* WV33^T^ (GCF 003076455.1).

To assess the relationships among bacterial strains via the identity/similarity values of the homologous regions of the target genomes [26,27], we analyzed the ANI values of 27 representative genomes of *Flavobacterium* strains, including *F. faecale* WV33^T^ (Table 4), using pyani [28] with four algorithms: a MUMmer (ANIm), BLASTN (ANIb), legacy BLAST (ANIblastall), and alignment-free algorithm tetranucleotide signature frequencies (TETRA) (https://github.com/widdowquinn/pyani, accessed on 6 July 2021) after retrieving whole genome sequences from the NCBI database (https://www.ncbi.nlm.nih.gov/refseq/, accessed on 14 May 2022). Statistical analysis of the 27 genomes showed an average size of 4,083,601 bp, with a minimum of 2,830,557 bp (*F. psychrophilum;* GCF_013343195.2) and a maximum of 6,096,872 bp (*F. johnsoniae;* GCF_000016645.1). The ANI values of the *F. faecale* WV33^T^ genome, compared against the genomes of the 26 related *Flavobacterium* strains, were 0.81–0.91 with ANIm, 0.70–0.91 with ANIb, 0.66–0.91 with ANIblastall, and 0.40–0.99 with TETRA (Figure 3), suggesting that strain WV3, 3^T^ was positioned in separate clades as a new species in the genus *Flavobacterium*, based on the commonly used ANIm, ANIb, and ANIblastall threshold values (<0.95–0.96, and the TETRA threshold value (<0.99) for species delineation) [29]. Notably, the *Flavobacterium* strains showed relatively broad ranges of ANI values, irrespective of the pyani algorithms used in this study, indicating that the *Flavobacterium* genomes are divergent in sequences.

### 2.5. Pangenome Analysis of the 27 Representative Genomes of Flavobacterium Strains

**Figure 4:**
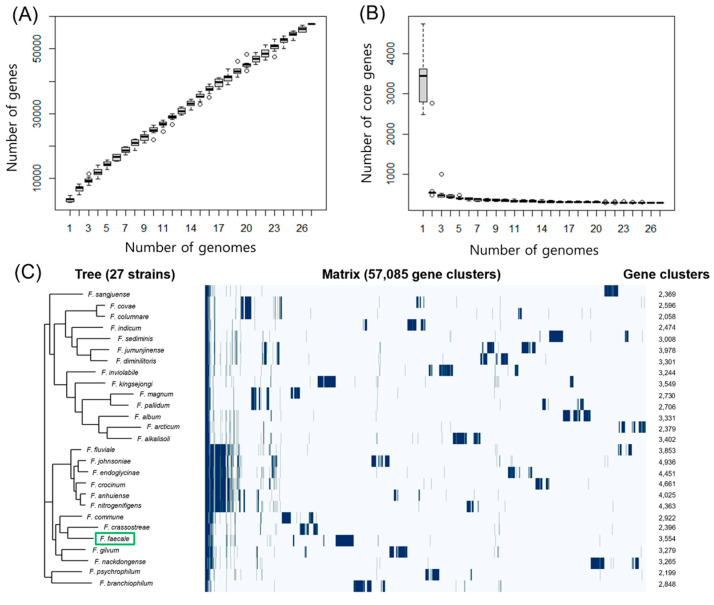
Pangenome analysis of 27 *Flavobacterium* strains including *F. faecale* WV33^T^. (**A**) Box-Whisker plot of pangenome as a function of the number of genomes (1–27) of *Flavobacterium* strains. (**B**) Box-Whisker plot of core genome as a function of the number of genomes (1–27) of *Flavobacterium* strains. Outliers are shown as open circles. (**C**) Phylogenetic tree and a gene presence/absence matrix plot generated using Roary. Green box represents *F. faecale* WV33^T^ (GCF 003076455.1). In the matrix a blue line represents gene presence. The numbers on the right represent the total number of core and unique genes of each *Flavobacterium* strain.

As the pangenome is regarded as the whole genomic repertoire of phylogenetically related microorganisms [30], pangenome analysis is frequently used to study the genomic diversity of microorganisms and discriminate the core, accessory, and unique genes present in pangenomes [20]. As 27 genomes of *Flavobacterium* strains are divergent in size (Table 4) and ANI values (Figure 3), it is worthwhile analyzing the pangenomes of the *Flavobacterium* strains to elucidate their genome evolution and variability. Therefore, the whole genome sequences of the 27 *Flavobacterium* strains were examined using Roary [31] with default parameters, except for the blastp identity cutoff value. In the range of 70–100% blastp identity cut-offs, the maximum number of clusters reached 85,318 at 95% identity cut-off, and the maximum number of core (conserved) genes was 294 at 80% identity cutoff (Table S2). The combined core (294) and soft core (57) genes (≥80% identity) were estimated to be 351, and the total gene clusters were 57,805 (Table S2), suggesting that *Flavobacterium* strains tend to be very plastic and have a small core genome. The number of pangenome genes increased steadily up to 57,805 with the addition of each further genome sequence (Figure 4A) and the number of core genes rapidly decreased and plateaued at 294 with the addition of a new genome (Figure 4B). In addition, a gene presence/absence matrix plot (Figure 4C) suggested that the *Flavobacterium*-type strains have a large and open pangenome.

### 2.6. Phylogenomic Analysis of the 27 Representative Genomes of Flavobacterium Strains

**Figure 5:**
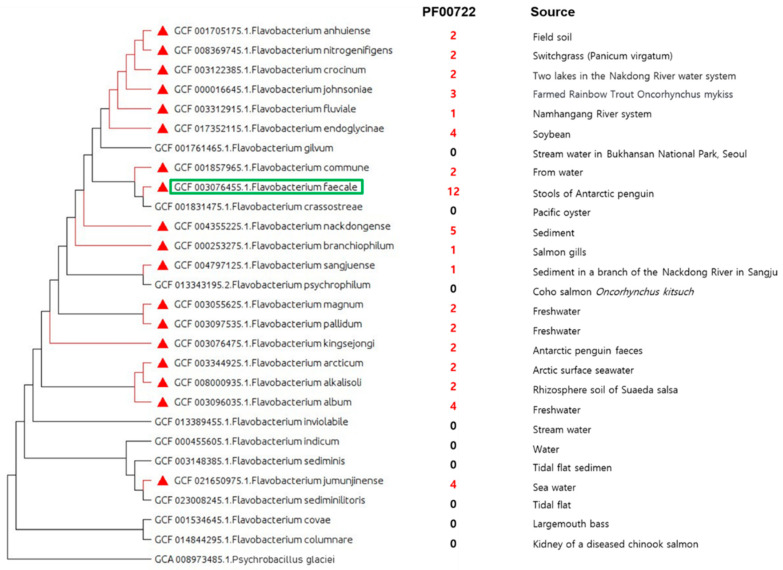
Phylogenomic analysis of 27 *Flavobacterium* strains including *F. faecale* WV33^T^. Phylogenetic tree was generated using GToTree and the red triangle represents the presence of predicted agarase (Pfam:PF00722). The numbers on the right represent abundance of Pfam:PF00722 (=GH16) in genomes of 27 *Flavobacterium* strains. Green box represents *F. faecale* WV33^T^ (GCF 003076455.1). *Psychrobacillus glaciei* (GCA_008973485.1) was used as an outgroup to root the tree.

Phylogenomics can reconstruct the evolutionary history of microorganisms at the genomic level with whole genomes or large fractions of genomes [32], and thus help infer the phylogenetic relationships of relevant microorganisms and gain insights into the molecular evolution mechanism [19]. Therefore, we analyzed the phylogenomics of 27 representative genomes of *Flavobacterium* strains (Table 4) using GToTree [33]. The phylogenomic analysis showed that *F. faecale* WV33^T^ was positioned close to *F. crassostreae* and *F. commune* (Figure 5), similar to the same position analyzed in the pangenome analysis (Figure 4C). We further analyzed the presence/absence of the genes encoding agarases across the 27 representative genomes of *Flavobacterium* strains with a protein family (Pfam) accession number (PF00722) (Figure 5) using GToTree. Pfam:PF00722 (https://pfam.xfam.org/, accessed on 6 July 2022) corresponded to the GH family 16 (GH16) of the agarase in the CAZyme classification (http://www.cazy.org/, accessed on 6 July 2022). Notably, 18 genomes contained GH16 (PF00722) at different abundance: the highest abundance (12 genes) was in *F. faecale* WV33^T^, followed by *F. nackdongense* (5 genes), *F. endoglycinae* (4 genes), *F. album* (4 genes), and *F. jumunjinense* (4 genes).

## 3. Discussion

The complete genome of *F. faecale* WV33^T^ consists of an approximately 4.6 Mb circular chromosome with a gene density of 885 genes/Mb. The genome size is similar to the average size of 4 Mb, with a minimum of 2.8 Mb (*F. psychrophilum*) and a maximum of 6.1 Mb (*F. johnsoniae*). Notably, [G] Carbohydrate transport and metabolism and [E] Amino acid transport and metabolism were relatively highly abundant in the COG category of the genome of *F. faecale* WV33^T^, similar to the other *Flavobacterium* strains. In particular, the abundance of [G] Carbohydrate transport and metabolism was further supported by the results of the CAZyme analysis (Table 3). *Flavobacterium* strains produce different types of carbohydrate-active enzymes, including polysaccharide-degrading enzymes. This is the case for *F. faecale* WV33^T^, the sequenced genome of which contains seven putative agarase-encoding genes. Transcriptional analysis revealed that five of these putative agarases were significantly enhanced by agar, suggesting that the putative agarases could be agar-metabolizing proteins. A biochemical and enzymatic study of the purified putative agarases of *F. faecale* WV33^T^ would elucidate the mechanism of agar-depolymerization. Among the 27 representative genomes of *Flavobacterium* strains, 18 genomes of *Flavobacterium* strains contained the GH16 family. Notably, *F. faecale* WV33^T^ contained 12 genes of GH16s, which was the highest abundance obtained in the 18 *Flavobacterium* strains. Therefore, *F. faecale* WV33^T^ could be used as a whole cell catalyst or microbial source for biocatalysts for the production of biomaterials of industrial importance.

Pangenome analysis revealed that *Flavobacterium* strains tend to be highly plastic and have a small core genome. Therefore, *Flavobacterium* strains are unique species with a small core genome and an open pangenome. This suggests that extensive gene gain/loss has occurred in this genus during evolutionary events. To address the extensive gene gain/loss events, additional studies, including mobile genetic elements, are required. Combined ANI, pangenome, and phylogenomic analyses showed that *F. faecale* WV33^T^ was positioned in a unique clade in the tree. As only 27 complete genomes of *Flavobacterium* strains were utilized in this study, a large-scale comparative genomic study with high quality and complete genomes of *Flavobacteria* can enhance the evolutionary information of *Flavobacterium* strains and our understanding of how the flavobacterial genome evolved to adapt to different environmental niches.

## 4. Materials and Methods

### 4.1. Strains and Culture Conditions

*F. faecale* WV33^T^ was aerobically cultured in Luria–Bertani (LB; 10 g/L tryptone, 5 g/L yeast extract, and 5 g/L NaCl) medium at 30 °C on a rotary shaker at 250 rpm. For transcriptional expression analysis of the putative agarases, *F. faecale* WV33^T^ was grown in LB medium supplemented with 1 g/L sliced solidified agar or 1 g/L glucose as a control.

### 4.2. Genome Sequencing and Assembly

Genomic DNA of *F. faecale* WV33^T^ was extracted using a genomic DNA extraction kit (Macrogen, Korea) with RNase A treatment. The genome of *F. faecale* WV33^T^ was sequenced in single-molecule real-time (SMRT) cells using Pacific Biosciences (PacBio) RS II SMRT sequencing technology (PacBio, Menlo Park, CA, USA). After the sub-read filtering of raw data, 78,126 long reads and 812,749,150 base pairs with a genome coverage of 176 folds were generated and assembled de novo using a Canu v1.3 assembler [34]. The overlapping regions at both ends of a contig were identified and trimmed to generate a unique stretch on both ends using Circlator [35]. Open reading frames (ORFs) were predicted by comparing the data obtained using the RAST server (https://rast.nmpdr.org/, accessed on 8 January 2020), Prodigal 2.6.3 [36], and Glimmer 3.2 [37] analysis tools. The tRNA and rRNA genes were predicted using tRNAscan-SE v1.21 [38] and RNAmmer v1.2 [39], respectively. Functional predictions were based on RPS-BLAST searches (E-value < 10^−3^) against the non-redundant GenBank protein database (www.ncbi.nlm.nih.gov/protein, accessed on 8 January 2020), clusters of orthologous groups (COG) database (www.ncbi.nlm.nih.gov/COG, accessed on 9 January 2020), and KEGG database (www.genome.ad.jp/kegg, accessed on 9 January 2020). A graphical circular map of the genome was constructed and visualized using Circos v0.67 [40].

### 4.3. Quantitative Reverse Transcription PCR

The total RNA of *F. faecale* WV33^T^ was extracted using the easy-BLUE^TM^ Total RNA extraction kit (iNtRON Biotechnology, Seongnam, Korea) and then treated with DNase I (Sigma-Aldrich, Saint Louis, MO, USA) at 37 °C for 30 min. The transcriptional expression levels of the five putative agarases of *F. faecale* WV33^T^ were determined using quantitative RT-PCR (RT-qPCR) using gene-specific primers (Table S1). Briefly, total RNA (1 μg) was subjected to cDNA synthesis using a ReverTra^TM^ Ace qPCR RT Kit (Toyobo, Osaka, Japan). The qRT-PCR was performed on a Rotor-Gene (Qiagen, Hilden, Germany) with a SensiFAST^TM^ SYBR No-ROX Kit (Bioline, Taunton, MA, USA), and the products were quantified using the comparative Ct (ΔΔCt) method. The gene encoding *gyrB* was used as the reference gene.

### 4.4. CAZyme Annotation

To analyze the CAZyme-related genes in *F. faecale* WV33^T^, the sequenced genome of *F. faecale* WV33^T^ was subjected to a FASTA format to run_dbcan3 v3.04, which is the standalone version of the dbCAN2 annotation tool [25].

### 4.5. Analysis of ANI

The 27 representative genome sequences of *Flavobacterium* strains were downloaded from NCBI (www.ncbi.nlm.nih.gov/refseq, accessed on 14 June 2022). Statistical analysis of the 27 genomes was performed using the statswrapper script in BBTools [41]. The ANI values of the 27 genomes of *Flavobacterium* strains were analyzed using pyani v0.2.7 [28] with four algorithms (a mummer [ANIm], blastn [ANIb], blastall [Ambilocal], and tetranucleotide signature frequencies [TETRA]) and default parameters.

### 4.6. Pangenome Analysis of Flavobacterium Strains Using Roary

The annotation files (GFF3) of 27 genomes of *Flavobacterium* strains were used for pan-genome analysis using Roary v3.11.2 [31] with a minimum blastp percentage identity of 70%, 80%, 85%, 90%, 95%, and 100%. The output files of Roary v3.11.2 were used to analyze and visualize the core and accessory genomes of *Flavobacterium* strains using R (https://www.r-project.org/, accessed on 14 June 2022).

### 4.7. Phylogenomics Analysis of Flavobacterium Strains Using GToTree

A phylogenetic tree of the 27 *Flavobacterium* strains was constructed using GToTree v1.6.34 [33]. Ninety single-copy gene sets of Bacteroidetes were used to construct the tree at the species level. *Psychrobacillus glaciei* (GCA_008973485.1) was used as an outgroup to root the tree.

## Figures and Tables

**Table 1:** General features and genome sequencing information of *F. faecale* WV33^T^ according to the minimum information about a genome sequence (MIGS) mandatory information.

Item ^1^	Description
General features	
Classification	Domain Bacteria Phylum Bacteroidetes Class Flavobacteriia Order Flavobacteriales Family Flavobacteriaceae Genus *Flavobacterium* Species *faecale*
Strain	WV33^T^
Gram stain	Negative
Cell shape	Rods
Motility	Non-motile
Pigmentation	Orange (zeaxanthin)
Temperature optimum	16 °C
Investigation	
Investigation type	*Bacteria*
Project name	Complete genome sequence of *Flavobacterium faecale* WV33^T^
Environment	
Geographic location	Antarctica
Latitude and longitude	62° 14′ 45.4″ S 58° 46′ 36.2″ W
Collection date	2011
Environment (biome)	Polar biome
Environment (material)	Antarctic penguin stools
Depth	NA
Oxygen requirement	Strictly aerobic
Pathogenicity	NA
Isolation and growth condition	PMID: 24893942
Sequencing	
Sequencing platform	PacBio RS II with P6-C4 chemistry
Assembly method	Canu v1.3
Number of replicons	1
Genome coverage	180×
Finishing strategy	Complete
BioProject	PRJNA383909
BioSample	SAMN06819498
Genomic features	
NCBI accession number	CP020918
Size (bp)	4,621,116
DNA G + C content (%)	35.2
CDSs	3984
tRNAs	67
16S-23S-5S rRNAs	18

^1^ Some features were obtained from a previous study [18].

**Table 2:** COG stat of genome features of *F. faecale* WV33^T^.

COG ^1^ Code	Function Category	Number of CDS ^2^
A	RNA processing and modification	1
B	Chromatin structure and dynamics	0
C	Energy production and conversion	134
D	Cell cycle control, cell division, chromosome partitioning	22
E	Amino acid transport and metabolism	170
F	Nucleotide transport and metabolism	55
G	Carbohydrate transport and metabolism	183
H	Coenzyme transport and metabolism	114
I	Lipid transport and metabolism	102
J	Translation, ribosomal structure, and biogenesis	151
K	Transcription	139
L	Replication, recombination, and repair	183
M	Cell wall/membrane/envelope biogenesis	260
N	Cell motility	6
O	Post-translational modification, protein turnover, chaperones	101
P	Inorganic ion transport and metabolism	205
Q	Secondary metabolites biosynthesis, transport, and catabolism	25
R	General function prediction only	289
S	Function unknown	232
T	Signal transduction mechanisms	126
U	Intracellular trafficking, secretion, and vesicular transport	27
V	Defense mechanisms	48
Z	Cytoskeleton	0

^1^ Clusters of Orthologous Groups of proteins; ^2^ Coding DNA sequences.

**Table 3:** CAZymes predicted in the genome of *F. faecale* WV33^T^.

	CAZyme Class *						
	GH	GT	PL	CE	CBM	AA	Total
Found in all 3 annotation programs **	88	48	10	3	5	0	154
Found in 2 annotation programs	13	26	1	1	3	0	44
Found in 1 annotation program	53	28	1	13	16	2	113

* GH, glycoside hydrolase; GT, glycosyltransferase; PL, polysaccharide lyases; CE, carbohydrate esterase; CBM, carbohydrate-binding module; AA, auxiliary activities; ** 3 annotation programs: HMMER, eCAMI, DIAMOND.

**Table 4:** Information of 27 representative genomes of *Flavobacteria* strains.

Assembly_id	Species Name	Size (bp)	Scaffold (n) *	N50	L50	N90	L90	GC (%)
GCF_003076455.1	*F. faecale*	4,621,116	1	1	4,621,116	1	4,621,116	0.351
GCF_023008245.1	*F. diminilitoris*	3,913,692	1	1	3,913,692	1	3,913,692	0.293
GCF_021650975.1	*F. jumunjinense*	4,694,567	3	1	4,671,850	1	4,671,850	0.304
GCF_017352115.1	*F. endoglycinae*	5,513,159	1	1	5,513,159	1	5,513,159	0.343
GCF_014844295.1	*F. columnare*	3,221,278	1	1	3,221,278	1	3,221,278	0.316
GCF_013389455.1	*F. inviolabile*	3,913,347	1	1	3,913,347	1	3,913,347	0.396
GCF_013343195.2	*F. psychrophilum*	2,830,557	2	1	2,827,614	1	2,827,614	0.325
GCF_008369745.1	*F. nitrogenifigens*	5,497,186	1	1	5,497,186	1	5,497,186	0.342
GCF_008000935.1	*F. alkalisoli*	3,985,855	1	1	3,985,855	1	3,985,855	0.379
GCF_004797125.1	*F. sangjuense*	3,130,338	1	1	3,130,338	1	3,130,338	0.360
GCF_004355225.1	*F. nackdongense*	4,217,227	1	1	4,217,227	1	4,217,227	0.362
GCF_003344925.1	*F. arcticum*	2,970,356	1	1	2,970,356	1	2,970,356	0.349
GCF_003312915.1	*F. fluviale*	4,839,571	1	1	4,839,571	1	4,839,571	0.338
GCF_003148385.1	*F. sediminis*	3,441,304	1	1	3,441,304	1	3,441,304	0.352
GCF_003122385.1	*F. crocinum*	5,877,431	1	1	5,877,431	1	5,877,431	0.339
GCF_003097535.1	*F. pallidum*	3,552,756	1	1	3,552,756	1	3,552,756	0.436
GCF_003096035.1	*F. album*	3,983,546	1	1	3,983,546	1	3,983,546	0.445
GCF_003076475.1	*F. kingsejongi*	4,224,053	1	1	4,224,053	1	4,224,053	0.397
GCF_003076455.1	*F. faecale*	4,621,116	1	1	4,621,116	1	4,621,116	0.351
GCF_003055625.1	*F. magnum*	3,464,207	1	1	3,464,207	1	3,464,207	0.469
GCF_001857965.1	*F. commune*	3,851,214	1	1	3,851,214	1	3,851,214	0.343
GCF_001831475.1	*F. crassostreae*	3,027,315	1	1	3,027,315	1	3,027,315	0.359
GCF_001761465.1	*F. gilvum*	4,402,594	1	1	4,402,594	1	4,402,594	0.351
GCF_001705175.1	*F. anhuiense*	5,109,718	1	1	5,109,718	1	5,109,718	0.343
GCF_001534645.1	*F. covae*	3,321,600	1	1	3,321,600	1	3,321,600	0.308
GCF_000455605.1	*F. indicum*	2,993,089	1	1	2,993,089	1	2,993,089	0.313
GCF_000253275.1	*F. branchiophilum*	3,563,292	2	1	3,559,884	1	3,559,884	0.328
GCF_000016645.1	*F. johnsoniae*	6,096,872	1	1	6,096,872	1	6,096,872	0.341

* All scaffolds were used in the analysis.

## Data Availability

The complete genome sequence of *F. faecale* WV33^T^ has been deposited in GenBank under accession number CP020918, and the strain has been deposited in the Korean Collection Type Culture (KCTC) under accession number KCTC 32457^T^.

## Supplementary Materials

The following supporting information can be downloaded at: https://www.mdpi.com/article/10.3390/ijms231810884/s1.

## References

[1] Bernardet J.F., Bowman J.P., Genus I., Krieg N.R., Ludwig W., Whitman W., Hedlund B.P., Paster B.J., Staley J.T., Ward N., Brown D., Parte A. (2011). Flavobacterium. Bergey’s Manual of Systematic Bacteriology.

[2] Touchon M., Barbier P., Bernardet J.F., Loux V., Vacherie B., Barbe V., Rocha E.P.C., Duchaud E. (2011). Complete genome sequence of the fish pathogen Flavobacterium branchiophilum. Appl. Environ. Microbiol..

[3] Huang L., Zhou J., Li X., Peng Q., Lu H., Du Y. (2013). Characterization of a new alginate lyase from newly isolated *Flavobacterium* sp. S20. J. Ind. Microbiol. Biotechnol..

[4] Bernardet J.F., Nakagawa Y., Dworkin M., Falkow S., Rosenberg E., Schleifer K., Stackebrandt E. (2006). An introduction to the family Flavobacteriaceae. The Prokaryotes, a Handbook on the Biology of Bacteria.

[5] Herrera L.M., Braña V., Franco Fraguas L., Castro-Sowinski S. (2019). Characterization of the cellulase-secretome produced by the Antarctic bacterium *Flavobacterium* sp. AUG42. Microbiol. Res..

[6] Lee C.C., Smith M., Kibblewhite-Accinelli R.E., Williams T.G., Wagschal K., Robertson G.H., Wong D.W.S. (2006). Isolation and Characterization of a Cold-Active Xylanase Enzyme from *Flavobacterium* sp.. Curr. Microbiol..

[7] Fu X.T., Kim S.M. (2010). Agarase: Review of Major Sources, Categories, Purification Method, Enzyme Characteristics and Applications. Mar. Drugs.

[8] Yang M., Mao X., Liu N., Qiu Y., Xue C. (2014). Purification and characterization of two agarases from *Agarivorans albus* OAY02. Process Biochem..

[9] Araki C.H. (1937). Acetylation of agar like substance of Gelidium amansii. J. Chem. Soc..

[10] Hamer G.K., Bhattacharjee S.S., Yaphe W. (1977). Analysis of the enzymic hydrolysis products of agarose by 13C-n.m.r. spectroscopy. Carbohydr. Res..

[11] Lau N.S., Tan W.R., Furusawa G., Amirul A.A.A. (2019). Complete genome sequence of the novel agarolytic Catenovulum-like strain CCB-QB4. Mar. Genom..

[12] Kirimura K., Masuda N., Iwasaki Y., Nakagawa H., Kobayashi R., Usami S. (1999). Purification and characterization of a novel beta-agarase from an alkalophilic bacterium, *Alteromonas* sp. E-1. J. Biosci. Bioeng..

[13] Wang J., Mou H., Jiang X., Guan H. (2006). Characterization of a novel beta-agarase from marine *Alteromonas* sp. SY37-12 and its degrading products. Appl. Microbiol. Biotechnol..

[14] Oh C., Nikapitiya C., Lee Y., Whang I., Kim S.J., Kang D.H., Lee J. (2010). Cloning, purification and biochemical characterization of beta agarase from the marine bacterium *Pseudoalteromonas* sp. AG4. J. Ind. Microbiol. Biotechnol..

[15] Morrice L.M., Mclean M.W., Williamson F.B., Long W.F. (1983). β-Agarases I and II from Pseudomonas atlantica. Purifications and some properties. Eur. J. Biochem..

[16] Fu W., Han B., Duan D., Liu W., Wang C. (2008). Purification and characterization of agarases from a marine bacterium *Vibrio* sp. F-6. J. Ind. Microbiol. Biotechnol..

[17] Macián M.C., Ludwig W., Schleifer K.H., Pujalte M.J., Garay E. (2001). *Vibrio agarivorans* sp. nov., a novel agarolytic marine bacterium. Int. J. Syst. Evol. Microbiol..

[18] Kim J.H., Choi B.H., Jo M., Kim S.C., Lee P.C. (2014). *Flavobacterium faecale* sp. nov., an agarase-producing species isolated from stools of Antarctic penguins. Int. J. Syst. Evol. Microbiol..

[19] Zhu Q., Mai U., Pfeiffer W., Janssen S., Asnicar F., Sanders J.G., Belda-Ferre P., Al-Ghalith G.A., Kopylova E., McDonald D. (2019). Phylogenomics of 10,575 genomes reveals evolutionary proximity between domains Bacteria and Archaea. Nat. Commun..

[20] Choi J.Y., Kim S.C., Lee P.C. (2020). Comparative Genome Analysis of Psychrobacillus Strain PB01, Isolated from an Iceberg. J. Microbiol. Biotechnol..

[21] Tatusov R.L., Galperin M.Y., Natale D.A., Koonin E.V. (2000). The cog database: A tool for genome-scale analysis of protein functions and evolution. Nucleic Acids Res..

[22] Mann A.J., Hahnke R.L., Huang S., Werner J., Xing P., Barbeyron T., Huettel B., Stüber K., Reinhardt R., Harder J. (2013). The genome of the alga-associated marine flavobacterium Formosa agariphila KMM 3901T reveals a broad potential for degradation of algal polysaccharides. Appl. Environ. Microbiol..

[23] Larsbrink J., Zhu Y., Kharade S.S., Kwiatkowski K.J., Eijsink V.G., Koropatkin N.M., McBride M.J., Pope P.B. (2016). A polysaccharide utilization locus from Flavobacterium johnsoniae enables conversion of recalcitrant chitin. Biotechnol. Biofuels.

[24] Kolton M., Sela N., Elad Y., Cytryn E. (2013). Comparative Genomic Analysis Indicates that Niche Adaptation of Terrestrial Flavobacteria Is Strongly Linked to Plant Glycan Metabolism. PLoS ONE.

[25] Zhang H., Yohe T., Huang L., Entwistle S., Wu P., Yang Z., Busk P.K., Xu Y., Yin Y. (2018). dbCAN2: A meta server for automated carbohydrate-active enzyme annotation. Nucleic Acids Res..

[26] Kim M., Oh H.S., Park S.C., Chun J. (2014). Towards a taxonomic coherence between average nucleotide identity and 16S rRNA gene sequence similarity for species demarcation of prokaryotes. Int. J. Syst. Evol. Microbiol..

[27] Paul B., Dixit G., Murali T.S., Satyamoorthy K. (2019). Genome-based taxonomic classification. Genome.

[28] Pritchard L., Glover R.H., Humphris S., Elphinstone J.G., Toth I.K. (2016). Genomics and taxonomy in diagnostics for food security: Soft-rotting enterobacterial plant pathogens. Anal. Methods.

[29] Yoon S.H., Ha S.M., Lim J., Kwon S., Chun J. (2017). A large-scale evaluation of algorithms to calculate average nucleotide identity. Antonie Leeuwenhoek.

[30] Tettelin H., Masignani V., Cieslewicz M.J., Donati C., Medini D., Ward N.L., Angiuoli S.V., Crabtree J., Jones A.L., Durkin A.S. (2005). Genome analysis of multiple pathogenic isolates of *Streptococcus agalactiae*: Implications for the microbial “pan-genome”. Proc. Natl. Acad. Sci. USA.

[31] Page A.J., Cummins C.A., Hunt M., Wong V.K., Reuter S., Holden M.T., Fookes M., Falush D., Keane J.A., Parkhill J. (2015). Roary: Rapid large-scale prokaryote pan genome analysis. Bioinformatics.

[32] Patané J.S.L., Martins J., Setubal J.C., Setubal J., Stoye J., Stadler P. (2018). Phylogenomics. Comparative Genomics.

[33] Lee M.D. (2019). GToTree: A user-friendly workflow for phylogenomics. Bioinformatics.

[34] Koren S., Walenz B.P., Berlin K., Miller J.R., Phillippy A.M. (2017). Canu: Scalable and accurate long-read assembly via adaptive k-mer weighting and repeat separation. Genome Res..

[35] Hunt M., Silva N.D., Otto T.D., Parkhill J., Keane J.A., Harris S.R. (2015). Circlator: Automated circularization of genome assemblies using long sequencing reads. Genome Biol..

[36] Hyatt D., Chen G.L., LoCascio P.F., Land M.L., Larimer F.W., Hauser L.J. (2010). Prodigal: Prokaryotic gene recognition and translation initiation site identification. BMC Bioinform..

[37] Delcher A.L., Harmon D., Kasif S., White O., Salzberphg S.L. (1999). Improved microbial gene identification with GLIMMER. Nucleic Acids Res..

[38] Lowe T.M., Eddy S.R. (1997). tRNAscan-SE: A program for improved detection of transfer RNA genes in genomic sequence. Nucleic Acids Res..

[39] Lagesen K., Hallin P., Rodland E.A., Staerfeldt H.H., Rognes T., Ussery D.W. (2007). RNAmmer: Consistent and rapid annotation of ribosomal RNA genes. Nucleic Acids Res..

[40] Krzywinski M., Schein J., Birol I., Connors J., Gascoyne R., Horsman D., Jones S.J., Marra M.A. (2009). Circos: An information aesthetic for comparative genomics. Genome Res..

[41] https://github.com/kbaseapps/BBTools.

