# Supplementary material for: Comparative Genomic Analysis of Agarolytic Flavobacterium faecale WV33T
Source: Int J Mol Sci. 2022 Sep 17;23(18):10884. doi: 10.3390/ijms231810884 (PMC9501601; doi:10.3390/ijms231810884)
Supplement: Supplementary file 1 [file ijms-23-10884-s001.zip › ijms-1901242-supplementary.pdf]

Table S1. Primers used in quantitative analysis of transcription levels of agarases

Gene	Sequence (5'→3')
gyrB	F: GGAAACTTTATTGGAGAGGT
	R: GTGTTGATGTTATTCACATAC
agar.3	F: GTATCCTAAAACTAATCACTAC
	R: ATCTGGACTCAACATCCATG
agar.2965	F: AAAAATGGGGTTACTGAAAGAA
	R: TTCTGGGCTAATGTAAAAACA
agar.3018	F: CCATCTGGTTTGGAAACCT
	R: AAATACATATCCTTGGTAAGTC
agar.3061	F: TAGAAAATGCAGTTACTGTCG
	R: ACCGCCACCCATTTCTGTT
agar.3068	F: TCGTTCAATTCGATGTTGATG
	R: TGGTAAAACTTCACAAAATCG

Table S2. Summary of pan-genomes with different Roary identity cut-offs

	Identity cut-offs (%)					
**Genes**	**70**	**80**	**85**	**90**	**95**	**100**
Core (99% <= strains <= 100%)	8	294	167	65	8	1
Soft core (95% <= strains < 99%)	1	57	24	13	1	1
Shell (15% <= strains < 95%)	964	2706	2393	1815	960	215
Cloud (0% <= strains < 15%)	84325	54748	62993	72893	84349	3538
Total (0% <= strains <= 100%)	85298	57805	65577	74786	85318	3755

.

Figure S1. Sequences of seven putative agarase genes present in the genome of *F. faecale* WV33^{T}

1. Agarase. 3

<Amino acid sequence>

MIYKFTIMKNKTFLVALLATSFQLLSCSKDSPEQPPAVVAPSPTPTPTPTPTPEVTDYKLIAVPADAGT
GKKWEFQTDFSDDFDYTYTPSTTKTTFGNGKWTNWYHNAWNGPGLTKWTYENVTIENGILKLITT
RVSGETKNYDYSGNTITGKATRLGAVVSNKQVVYPVYIESRIKIANSVNTSGAWMLSPDDTQEIDFM
EAWGGKQARNNVSGGRQFSKTIHLSHHVFIRTPFQDYQPGDVSTWFTDSKVGDWSDAYHRYGVF
WKSPTELEYYIDGVLVRSTNSLDNVGTKDGMDPKNFTSPTKEAATRTGLSKPMDILFTMEDQNWR
AGIGCTPTDEEIKDTDGHTMKIDWIRIFKPVTK

<Nucleic acid sequence>

atgatttataaatttactatcatgaaaaacaaaacttttctagtagcattattagcaacatcatttcagttgctgtcgtgttcaaaagattctccagagcaa
cctcctgcagtagttgcaccaagtccgacacccaccccaacacctacacctacaccagaagtaacagattacaaactaatcgctgtacctgctgatgct
ggaacaggaaaaaaatgggaatttcaaactgacttttcagatgattttgactacacctatacgccatctacaaccaaaacaacttttggtaacggaaa
atggaccaactggtatcataatgcatggaacggaccaggattaaccaaatggacatatgaaaatgttactattgagaatggtatcctaaaactaatca
ctacgcgtgtaagcggtgaaacaaaaaattatgattacagcggtaacaccatcacaggcaaagcaacacgcctaggtgctgttgtctccaacaagc
aagttgtctatccagtatatatagagagtagaattaaaattgcaaacagtgtcaatacctctggagcatggatgttgagtccagatgacacacaagaa
atcgatttcatggaagcttggggtggtaaacaagctagaaataacgtaagtggcggaaggcaattttcgaaaacaattcacttaagccaccacgtat
ttattcgtactccttttcaagattaccaacctggcgatgtgtctacttggtttacagatagcaaagtaggtgattggtcagacgcctaccatcgttatgga
gtattctggaagagccctaccgaactagaatactatatcgatggcgttttggttcgttctacaaatagcttggacaatgtaggaaccaaagatggaat
ggacccaaaaaacttcacttcgcctacaaaagaagcagcaactagaacaggcttaagcaaaccaatggacattttgttcaccatggaagatcaaaac
tggagagccggaattggttgcacacctactgatgaagaaattaaagataccgacggccatacaatgaaaattgactggattcgaatattcaaaccg
gttaccaaataa

2. Agarase.162

<Amino acid sequence>

MGTWVLIPELSNEFNYSSKTDPAFTTDWKDSYINSWTGPAPTKWVASGSSLGTAPTGEKCIILQATK
VGNTLECGMVTSKAKATFPLYQEARVRISDSHLANAVWMLSTDSREEIDNLESYGDKLGSKPYFGD
KLHLSHHTFKDIAGIRKDYQPQLETWMIRKTGTGCNATDDVKWNTDFHIFGVKWVDATNLEYYV
DGVKVRTVTGTDIDPNGYTDCSGMKKALHMIISQAAQTWRFADVNEFYNQNITNGARTKMYIDW
IRVYRPNQNLGTSTCN

<Nucleic acid sequence>

atgggcacttgggttttaatacctgagctctcgaacgaatttaattattcaagcaaaactgatcctgcgtttacaacagactggaaagactcttacatta
atagttggacaggaccagcaccaacaaaatgggtcgctagtgggtcttcacttggaacagcaccaactggagaaaaatgtataattcttcaagcaac
aaaggtggggaataccttagaatgcggaatggttacatctaaagctaaagcaacattcccactttaccaagaggccagagtaagaataagtgattct
cacttagccaatgccgtgtggatgttaagtactgattctagagaagaaattgacaaccttgaatcttatggagataaattagggtcaaaaccatatttg
gagataaattacatttaagccatcatacttttaaagatattgcaggtattagaaaagattatcaaccccaattagagacctggatgataagaaaaacag
ggacgggttgcaatgctactgatgatgtaaaatggaataccgatttccatatttttggggtaaaatgggttgatgctactaatctagaatattatgtaga
tggagtaaaagtaagaaccgtaacaggcaccgatattgacccgaacggctacacagattgttcaggaatgaaaaaagctttgcatatgattattagt
caagcagcacaaacatggcgatttgctgacgtgaatgagttttacaaccagaacattaccaatggcgcaagaactaagatgtacatagactggattc
gtgtttacagaccaaatcaaaatttaggcacctctacttgtaattaa

3. Agarase.2965

<Amino acid sequence>

MFQLTLKNQIMKLKKLQKISLGLLTVFFTVSTIVAQPAAPSGKKWVKATYLSDDFNGSFDTSKWEQ
SIWNYAGTPTLMNNNNSGVSGGNLWIKATDNGQSNSGAWFQSSRVQSKAKVGFPMYTECRIKAA
NISAYSTYWLNNGDANNRDEIDICENNPKPSITSQTYRPYTMYSQYFIVKNGVTERNAGNFDNRNL
PAGNPAKGVKWNTYQVLGCYWKDSKHVQFYINGAPAGSLTTTGVFTLAQNIIWDLWTGPFDYLG
GLAVRSDLANEAQSTMYVDWINTYTLANITGKEVESKLKSETYNYKTSVSPNPVSNLLQVTLKSEN
DYNSYSIIDPLGRSVAQGKIAVGSAEYNVNVSSLKSGIYFLILNGEDDSEKIKIMKN

<Nucleic acid sequence>

Atgtttcaattaaccttaaaaaaccaaattatgaaattaaaaaaattacaaaaaatcagtttaggactgttgacagtattctttactgttagtactattgtt
gcacaaccagccgccccttctggaaaaaagtgggtaaaagctacatatctttcagatgattttaacggatcctttgatacttcgaaatgggaacaatct
atttggaattacgcaggaacaccgactcttatgaataataacaattcgggtgtctcaggtgggaatttatggatcaaagcgacagataatggacaaa
gtaactcaggagcgtggtttcagtcttctcgagtacaatctaaagcaaaagtagggtttcctatgtacactgaatgccgaattaaagcggctaatatttc
tgcttattctacttattggttaaataatggggatgcaaataaccgtgatgaaattgatatttgcgagaataatcccaaaccttcaatcactagtcagacat
acagaccttacacaatgtattcacaatatttcatagttaaaaatggggttactgaaagaaatgctggtaattttgacaatagaaacctccctgctggtaa
tcctgcaaagggagtaaaatggaatacttatcaagttcttggctgctattggaaagattctaaacatgtacaattttacattaatggagcgccagcggg
tagtttgacaaccactggtgtttttacattagcccagaatattatttgggatttatggacaggtccatttgattatcttggtggccttgcagtaagaagcg
atttggcaaatgaggctcaaagtacgatgtatgtagattggataaatacatatacattggctaatattacaggaaaagaagtcgagtctaagcttaaa
tcagaaacttataattacaaaacttctgtgtcaccaaatccagtttcgaatttattgcaggtaaccttaaaatcagaaaacgattataattcctatagtatt
atagatcctttaggtagatcagtagcgcaaggtaaaattgcagtcggatcagcagagtacaatgtaaatgtatcatctttaaaatctggaatctatttttt
aattttaaatggagaggatgattcggaaaaaattaagattatgaagaattaa

4. Agarase.3018

<Amino acid sequence>

MFITFSIVIAAACTKDEAPAPTPKATSISTKTPVGATLYSEIETGGTITLGNDNLTITERGICWGETTNP
TTATNKKITTSNSNTFDLTLTGLKENQIYYLKAYFVAGSVTFYGDQKQLTTAKKPSEVSFLPSAGNN
KSWVIQPVFSNEFNYDGKTADFTTNWQDRFFNGWTGALPTVYSASQSTITGGELVYKATIEKKSTG
DIIRTGIVTSKTKVGYPMYMEARVKISESSLASAVWMLSEDSTQEIDNLEAYGDKTNDYYSKRLHLS
HHVFIRTPFQDYQPSGLETYYADGKGTQWANDYHNYGVLWLDPFTLKYYVDGVLVRTTPINQIDP
NNYAGGTGLTKDMYLIISAASQPWRQSQGINYFTDPSVLDDARSTMRVDWIRVYKPE

<Nucleic acid sequence>

atgttcattactttttctattgtcatagcggctgcatgtacaaaagacgaagcaccagcgccgactcccaaagctacttccattagcaccaagactcctg
tgggcgcaactttatattctgaaattgaaacaggaggaaccattactttaggaaatgacaatttaacaattaccgaaagaggaatctgctggggaga
aaccaccaacccaactacagcaaccaataaaaaaataactactagtaattccaatacatttgatctcactttgacaggattaaaagaaaaccaaatcta
ttacctaaaagcctattttgtagctggttccgtaactttttatggtgatcaaaaacaacttacaactgccaaaaaaccaagcgaagttagttttctaccaa
gtgctggaaacaataaaagctgggttattcaacctgttttctccaatgagtttaattatgatggaaaaacagccgattttaccaccaattggcaagacc
gtttcttcaatggatggacaggagctcttccaactgtatattctgcttctcaatcaaccattactggtggcgaattagtctacaaagctaccatagaaaaa
aaatcaactggagatataattcgaactggaatagttacgtctaaaactaaagtcggttatccaatgtatatggaagcacgagtgaaaattagcgaatc
ttcattagcttcagctgtttggatgctcagcgaagactcaactcaggaaattgacaatctagaagcttatggcgataaaacgaatgattactactccaa
acgtttacacttaagtcatcatgtcttcataagaacaccatttcaggattaccagccatctggtttggaaacctattatgctgatggcaagggaacacaa
tgggcaaacgattaccataattatggagtattatggttagatccttttacattaaaatattatgtagatggtgtactagtaagaacaacgccaatcaacc
aaattgaccctaataattatgctggtggaactggacttaccaaggatatgtatttaattatttcggccgcatcccaaccctggagacaatcacaaggca
taaattactttactgatccatccgtgttggatgatgctcgatcaaccatgcgcgtagactggattagagtttataaaccagaatag

5. Agarase.3061

<Amino acid sequence>

MKKNILFIAGTFLTFSSLLSAQIKAPEGKSWQLVSELSDEFNGKKLDTKKWIADPEGHPEFGWIGRSP
ALFIENAVTVENGYLNIEVGKLDERFVSNKYNTATNYDYYGGIIRAVKPVTYGYYFESTFKMSKTEM
GGGFWIMSKNTCGKKHEIDITESVGSISPLAQEWGKKWDKIMHSNTILRNTTCNEEKRDQGMILPE
TKNSEKFYTYGCWWKSPNELLFYLDGKYVVYTLTPPADFDQQMFIHFSIEVYDWNPIPEKDSKLMTA
SKKDRTALIDYIRTYKLVTVKP

<Nucleic acid sequence>

atgaaaaaaaacattctttttatagctggaactttttttaacatttagttctctattaagtgcgcaaattaaagcgcctgaaggtaaatcatggcaattggtt
tctgaattatcagatgagtttaacggaaaaaaattagataccaaaaaatggattgcagatccagaaggtcatcctgaattcggttggattggtcgttct
cctgcccttttttatagaaaatgcagttactgtcgaaaacggatatttaaatattgaagttggaaaactagacgagcgttttgtaagcaacaaatataata
cggctaccaattatgattattacggaggtatcattagagcagtgaaacctgtaacttatggttattattttgagagtactttcaaaatgagcaaaacaga
aatgggtggcggtttttggataatgtctaaaaatacctgtggcaaaaaacacgaaattgacattaccgaatctgtcggaagtatttctccactagcaca
agaatggggcaaaaaatgggataaaattatgcattccaataccatttacgaaatactacttgcaacgaagagaaaagggatcagggaatgatcctt
ccagaaacaaaaaaattctgaaaaattctatacctacggttgttggtggaagagcccaaatgaattattattctatttggatggaaaatatgtgtatacac
taacacctcctgcggactttgaccaacaaatgtttattcatttttcaattgaagtctacgattggaatccaattcctgagaaagacagtaaactgatgaca
gcatccaaaaaagacagaactgccttgattgactacataagaacctataaattggttacggtaaaaccttaa

6. Agarase.3068

<Amino acid sequence>

MKKQYLILLLVPAAFFAQVKVAVNLNVKHTVGGVSTFERSKFITMHSNTTDTEWDGDNQIGDLRN
NFLNGLDVYLGRETGLITYTLNQVAQDPARAGYASPTDIASRGLSSRNSFAAKTNLAQYESRKGGM
IFGGQLYPFWTGVGQKPTAKGWNIANGTATGEYMGRFMKEFFGGNGPSLPAFLEIVNEPDYELLG
GVKEYTKTIAEIANFHIAAADAIRAQMPNTKICGYTNSFPYYEVGEFQRWFNRDKMFMDMAGSKM
DYYSIHLYDFPTIGGKRQMRSGSNLEATFDMMDHYGMLSVGSVKPYVLSEYGAQTHDSNNQQWSS
YRDWLNVKSFNSMLMSFLERPNTIASAIPFVIVKAEWGYNSTTGVPYTSRLMRKANEPASYTGQWV
YTDFVKFYQQWKNVKGTRVDIKSDDPDILVKAYVDGNKGYVIINNLEQAVKTIDLNLFDHDANNL
TQIVKRYLTLTANQTDLQETTTTSPITSLTIGSESHVVLEYTFANPIAIDRTLDETKYYADDYLKPIVAA
NTPVVYNVNGITKATYGEAILRIGVGRATGKSLVPVIKVNNTLVTTPTDFRGDNQPQRDSFFGVLEI
PVPYNLLDVNNVISVQFPDAGGNVSSVCMQVNNFSHNIRVLATNTFKYDSQISLAPNPASDMVTL
KIVEELIGSSASIYNMQSALVQTEKLSQLDNNFTIAKLPKGVYFVVLKKNNTTMAKVKLIVN

<Nucleic acid sequence>

atgaaaaaacaatacctaatacttcttttagtgccggctgcttttttttgcccaagtaaaagtagctgtgaacttgaatgtcaaacatactgttggtggcgt
ttcgacttttgaacgctctaagtttattacgatgcactctaatacaacggatacggaatgggatggagacaatcaaataggtgatctacgaaataatttc
ctaaacggactggatgtttatttgggtcgtgagacgggattgattacgtatactttgaatcaggtggcacaggatcctgcacgtgctggttatgcaagt
cctactgatattgcttcaagaggattgtcatctcgaaatagttttgctgctaaaaccaatttagcgcaatacgagagccgcaagggaggaatgattttt
gggggtcaattgtatccatttttggacgggtgttgggcaaaaaccaacagctaaaggatggaatatagccaacggaactgctacaggagaatacat
gggacgttttatgaaagaattttttggagggaacggacctagtttacctgcttttttggaaattgtcaacgagcctgattatgaactattgggtggtgtg
aaagaatataccaaaacgattgcggagatagccaattttcacattgcagcagccgatgcaattagagcgcaaatgccaaatactaagatttgtggct
ataccaattcgtttccgtattacgaagtaggggagtttcaacgttggtttaaccgagacaaaatgtttatggatatggcaggaagcaaaatggattatt
attcgattcatttatatgatttccaactattggcggaaagcgtcagatgcgttctgggagtaatcttgaagccacatttgacatgatggatcattatggt
atgctttcggtagggtcggtaaagccttatgtgctttcggaatatggagcacaaacacatgattcaaacaaccaacaatggagttcgtacagagattg
gctaaatgtgaaatcgttcaattcgatgttgatgtcgtttttagaaagacccaacacaattgctagtgcgattccgtttgtcatcgtaaaagcagaatgg

ggttacaacagcacaacgggagttccttatactagccgattaatgcgcaaggccaatgagccagcgagttatacaggacaatgggtttataccgatt
ttgtgaagttttaccaacaatggaaaaatgtaaaaggaactcgagtagacatcaaatcagacgatcccgatattttagttaaagcttacgttgatggca
acaaaggatatgtgattataaacaatctagagcaagcagttaaaacaattgatttaaatttatttgatcatgatgctaataacttaacgcaaattgtaaa
acgttatttgaccttgacggccaaccaaacagatttacaagaaacaacaacaacttctccgattacgtccttgactatcggttcagagtcacatgttgttt
tagaatatacttttgccaatcctattgcgattgatagaaccttggacgaaacaaaatactatgccgacgactacttaaaaccaattgttgcagcaaatac
gccagtggtttataatgtaaacggaataacgaaagcaacttatggcgaggctattttgcgtattggggtgggtagagcaaccggaaaatcactggtt
cctgtaatcaaagtgaataatacccttgtcacaactccaaccgattttagaggggataatcaacctcaaagagattctttttttggagttttagagattcct
gtgccctacaatcttttggatgtgaacaatgtaatttcagttcagtttccagacgcgggcggaaacgtgagtagtgtatgtatgcaagtaaataattta
gtcataatattcgagtattagcaaccaacacatttaaatatgattcgcaaattagtttagcaccaaatcctgctagcgatatggtaacgttaaaaattgta
gaagaattgattggcagttctgcaagtatttacaatatgcaatcggctttagttcaaacagaaaaactaagtcagttagataataatttcacaattgcta
agttgccaaagggagtttattttgtagtactaaagaaaaataatacaacgatggccaaagtgaagttgatcgtaaattag

7. Agarase.3154

<Amino acid sequence>

MKATLLSFIALSSLILSCTNKEESTLLESASIFDDNLISQNLTNKSILSTNCPKPADANLFIAITNNNSN
VLNRSQNGMGTWVLIPELSNEFNYSSKTDPAFTTDWKDSYINSWTGPAPTKWVASGSSLGTAPTGE
KCIILQATKVGNTLECGMVTSKAKATFPLYQEARVRISDSHLANAVWMLSTDSREEIDNLESYGDK
LGSKPYFGDKLHLSHHTFKDIAGVRKDYQPQLETWMIRKTGAGCNATDDVKWNTAFHIFGVKWV
NATNLEYYVDGVKVRTVTGTDIDPNGYTDCSGMKKALHMIISQAAQTWRFADMNEFFNQNITNG
ARTKMYIDWIRVYRPNQNLGTSTCN

<Nucleic acid sequence>

atgaaggcaacattattatcttttatcgctttatccagcttaatactaagctgtactaataaggaagaaagcacattattggaatcagcgtcaatatttga
cgacaatttaatttctcaaaaccttaccaacaaatccattctttctacaaattgtcctaaacctgcagacgctaatttatttatcgcaattaccaacaacaac
tccaatgtgttaaatcgttcccaaaacggcatgggcacttgggttttaatacctgagctctcgaacgaatttaattattcaagcaaaactgatcctgcgtt
tacaacagactggaaagactcttacattaatagttggacaggaccagcaccaacaaaatgggtcgctagtgggtcttcacttggaacagcaccaact
ggagaaaaatgtataattcttcaagcaacaaaagtggggaataccttagaatgcggaatggttacatctaaagctaaagcaacattcccactttacca
agaggccagagtaagaataagtgattctcacttagccaatgccgtgtggatgttaagtactgattctagagaagaaattgacaaccttgaatcttatg
gagataaattagggtcaaaaccatattttggagataaattacatttaagccatcatacttttaaagatattgcaggtgttagaaaagattatcaacccca
attggagacctggatgataagaaaaacaggggcgggttgcaatgctactgatgatgtaaaatggaataccgctttccatattttggggtaaaatgg
gttaatgctactaatctagaatattatgtagatggagtaaaagtaagaaccgtaacaggcaccgatattgacccgaacggctacacagattgttcagg
aatgaaaaaagctttgcatatgattattagtcaagcagcacaaacatggcgatttgctgacatgaatgagtttttcaaccagaacattaccaatggcgc
aagaactaagatgtacatagactggattcgtgtttacagaccaaatcaaaatttaggcacctctacttgtaattaa
